# Secondary penile tumours revisited

**DOI:** 10.1186/1477-7800-3-33

**Published:** 2006-10-11

**Authors:** Jacob Cherian, Sreekumar Rajan, Ali Thwaini, Yaser Elmasry, Tariq Shah, Rajiv Puri

**Affiliations:** 1 Department of urology, Bradford teaching hospitals, Bradford, UK; 2 Department of urology, Barts and Royal London NHS trust, London, UK

## Abstract

**Objective:**

To highlight the salient features of metastatic malignancies involving the penis, with special reference to the primary tumour sites, metastatic mechanisms, clinical features, differential diagnosis, treatment and prognosis.

**Methods:**

A comprehensive search of the literature was performed using MEDLINE and EMBASE, using the keywords 'penis', 'secondary malignancy', 'metastasis' and 'malignant priapism' to identify reviews and case reports of secondary penile malignancy. A case of rare clinical presentation of metastatic penile lesion is presented along with the review of the literature.

**Conclusion:**

Secondary malignancy of the penis is a rare clinical entity, despite the rich vascularisation of this organ. The majority of metastatic lesions take their origin from the neighbouring genito-urinary organs, mainly prostate and bladder. These lesions are often associated with disseminated malignancy and hence have a poor outcome. Nodular or ulcerative lesions involving the corpora cavernosa or priapism are the main modes of clinical presentation. In most cases, only palliative or supportive therapy is indicated.

## Background

Metastatic involvement of the penis is relatively infrequent, compared to its primary counterpart, despite rich vascularisation and extensive circulatory communication between the penis and the neighbouring organs. The vast majority of the primary lesions are in the genitourinary organs, with the recto sigmoid region contributing to the bulk of the remainder [1]. Penile involvement is usually associated with disseminated disease and generally portends a poor prognosis [2]. We report an unusual presentation of penile secondary from a rectal primary and a brief review of literature.

## Case report

**Figure 1:**
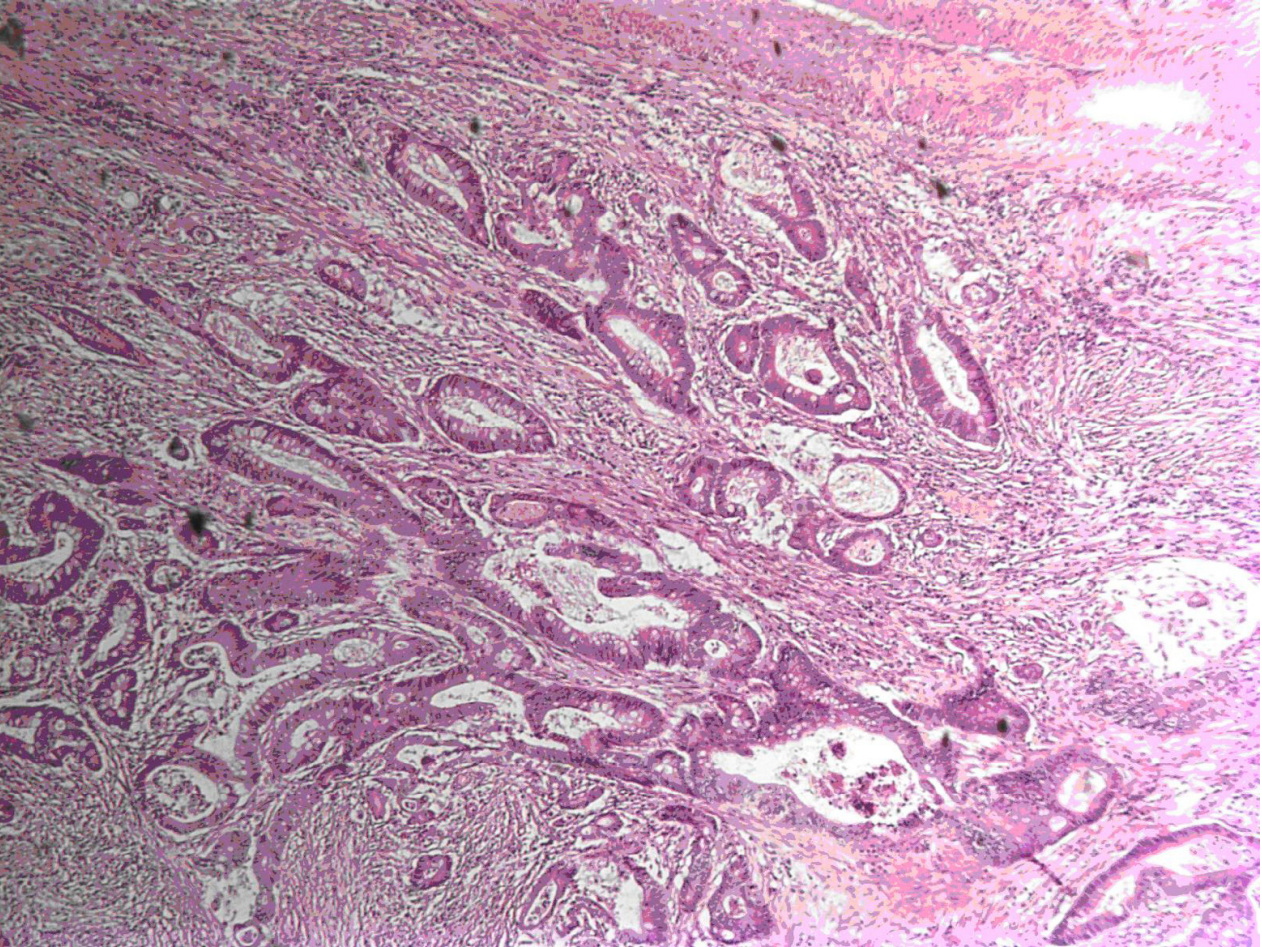
h & e section; infiltrating rectal adenocarcinoma, reduced from ×50.

**Figure 2:**
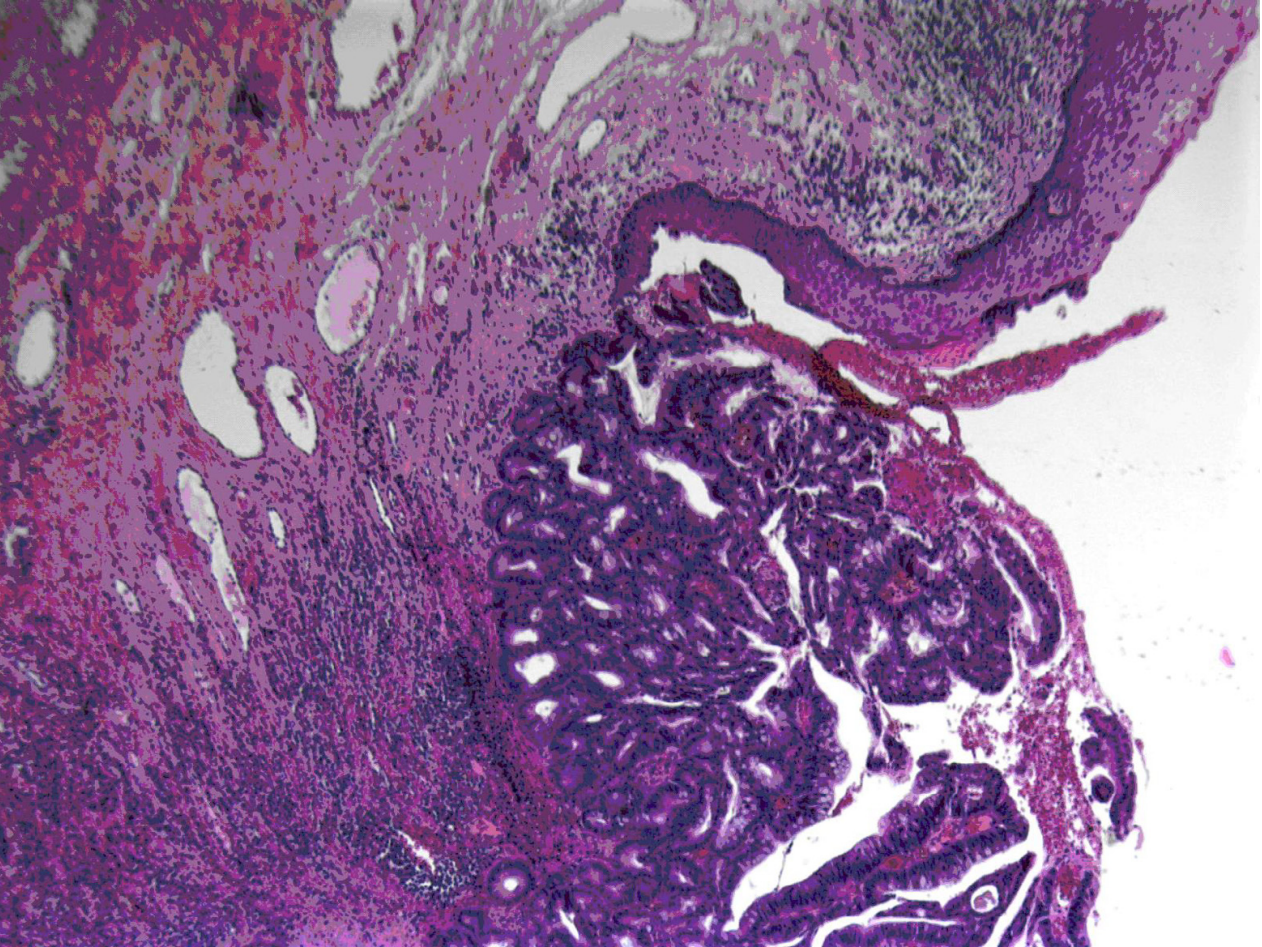
h & e section; metastatic adenocarcinoma deposit with surface ulceration and adjacent squamous epithelium, reduced from ×25.

A 73 year old man was referred with complaints of a painless lesion under the foreskin and penile discharge. Five years prior, he had undergone abdomino-perineal resection for Duke stage-B adenocarcinoma of the rectum (Fig 1). Six months later, he developed recurrence in para-aortic lymph nodes and liver and was started on combination chemotherapy. In spite of continuing chemotherapy, he developed further recurrence locally as well as in the lung and lower rectus muscle. At presentation, retraction of the foreskin revealed a 1.5 cm non-tender ulcero-proliferative lesion on his foreskin. There was a second 0.5 cm nodular lesion on the prepuce, separate from the first. Both the lesions were confined to the foreskin, without any involvement of the glans or the corpora. There was no oedema of the prepuce or the skin of the penile shaft and no other palpable lesions on the corpora or glans. The superficial inguinal lymph nodes were not significantly enlarged. Excision biopsy by circumcision revealed metastasis from adenocarcinoma of rectum (Fig 2). The patient was not offered further specific therapy because of the advanced and progressive nature of his disease and eventually succumbed to it in four months time.

## Discussion

Even though penile metastatic lesions are generally infrequent, it should always be considered in the differential diagnosis in any patient presenting with a penile lesion or priapism, in the setting of a disseminated malignancy. Penile secondary lesions commonly affect the shaft or glans penis and preputial lesions when present usually accompany the former. Isolated secondary deposits involving the foreskin alone, like the case presented above, are extremely rare. To our knowledge, there is only one previous report in the literature, of isolated metastatic lesion on the prepuce [3].

Most penile metastases are associated with disseminated malignancy and thereby portend a poor prognosis [4]. About 75% of the metastasis to the penis originates from primary malignant tumours in the neighbouring urogenital organs [1,2]. Recto sigmoid cancers constitute around 13% of them [1].

### History

The earliest report of secondary penile malignancy is credited to Eberth in 1870, when he reported metastasis from an adenocarcinoma of the rectum [5]. Two years later, Roberts noted the first case of penile secondary from a genitourinary primary [6]. Subsequently there were many reports of metastasis to the penis from a wide range of primary sites, totalling to over 370 reported cases to date. In 1956, Paquin and Roland reported nine new cases and postulated the various possible mechanisms of spread of tumour to the penis [7]. Abeshouse et al presented a comprehensive review on the subject in 1961, when they summarized all the 140 cases until date [2]. Further informative reviews were written by Hayes and Young [8] and Weitzner [9], identifying 165 cases until 1971. More recently, Osther et al (1991) published a detailed review on this topic revisiting the metastatic mechanisms [4]. Perez et al (1991) reviewed the literature and tabulated all the 307 cases reported thus far, based on the primary site of involvement [1]. Since then, another 65 cases have been reported, excluding our current case (Table 1).

**Table 1:** Site specific list of all reported cases of secondaries to penis (total 372; 65 new cases added on to 307 reported until 2000 by perez et al ^1^)

Primary site	New cases since 2000	Total number (per cent)
Prostate	32	125 (34)
Bladder	15	112 (30)
Recto-sigmoid + Rectum	8 (Rectum 7, Recto-sigmoid 1)	48 (13)
Kidney	1	30 (8)
Lower GI tract (excl. Rectum & ecto-sigmoid)	4	15 (4)
Testis	1	11 (3)
Lung	1	4 (3)
Upper airway	-	4 (1)
Upper GI tract	1(Stomach)	3 (0.8)
Bone	-	3 (0.8)
Ureter	-	2 (0.5)
Hepato-biliary	-	2 (0.5)
Others	1(Urethra)	6 (2)

Total	65	372

### Primary tumours

The vast majority of secondary tumours in the penis take their origin from the genitourinary organs within the pelvis. Previously bladder has been reported as the commonest primary site [1,2], but a review of the literature since 1990 revealed a larger number of reported cases from prostatic primary, making it the commonest now. The other common primary tumours include the kidney, testis, recto-sigmoid, rectum and colon. There are isolated reports of metastasis to the penis from upper gastrointestinal tract, lung, skin and osseous primaries. A break up of all the primary sites is presented in Table 1.

### Metastatic mechanisms

The rarity of metastatic involvement of the penis has been a clinical enigma because of its rich vascularity and being an end organ with respect to arterial, venous and lymphatic systems. Paquin and Roland in 1956 first described the possible mechanisms by which tumour spreads to the penis [7]. It is very difficult to elucidate the exact mode of spread in individual cases, as penile secondaries are usually associated with disseminated disease. The five most accepted mechanisms of spread are listed below.

#### a. Retrograde venous route

This is presumed to be the commonest mode of spread to the penis [2,7]. The established communications between the dorsal venous system of the penis and the venous plexuses draining the pelvic viscera provide routes for easy transportation of malignant cells. This route of spread can explain the majority of secondary tumours arising from the prostate, bladder and the recto-sigmoid. This would also account for the localisation of the vast majority secondary lesions on the corpus cavernosa and the glans of the penis. Reversal of flow in these venous channels facilitates direct access to the penis, for the pelvic venous blood. Permanent reversal of flow through the communicating venous channels can occur when there is blockage more proximally, either by tumour cells within them or by extrinsic pressure. Alternatively, there can be intermittent episodes of retrograde flow, during phases of raised intra-abdominal pressure, like coughing or sneezing [2].

#### b. Retrograde lymphatic route

The mechanism for this spread occurs in a manner similar to the retrograde venous route [7]. The lymphatics from the penis, as well as those from the bladder base and the posterior surface of the prostate, drain into the external iliac nodes. Similarly, lymphatics from the lower rectum pass through the perineal region into the inguinal nodes and then to the iliac nodes. These connections provide an excellent route for the tumour spread along these vessels, either by permeation or as emboli. This is probably the route of spread occurring primarily to the penile skin, rather than the corpora or the glans penis.

#### c. Arterial spread

This mode of spread of tumours to the penis is uncommon, perhaps explaining the rarity of sarcomatous secondaries in the penis [2]. There are three possible ways in which tumour cells can gain access to the arterial circulation. These include direct tumour extension into the arterial pathways (eg. branches of hypogastric artery), secondary tumour emboli (originating from secondary deposits in the lung) and tertiary embolism (emboli from a metastatic liver lesion producing lung lesions, from which emboli reach the penis) [7].

#### d. Direct extension

This mode of spread is possible from some highly invasive primary tumours of the prostate and the bladder, which enjoy close anatomical relationship with the penis [7]. In addition, tumour cells from an aggressive low-lying rectal carcinoma can spread along the ischi-rectal fossa, onto the base of the penis. However, this could only account for lesions involving the more proximal parts of the penile shaft, which interestingly is much less common than discrete lesions of the distal corpora and glans.

#### e. Implantation and secondary to instrumentation

These have been described as possible mechanisms for tumour spread to the penis even though they appear highly unlikely considering the fact that isolated lesions of the corpus spongiosum, without concurrent involvement of the corpora cavernosa or the glans, are practically non-existent [7].

### Clinical presentation

Patients with penile secondaries generally have widespread metastatic disease and thereby poor general health. However there are reports of delayed metastatic involvement of the penis, years after the primary has been treated, and without evidence of any other metastatic lesions [10]. The mean age of presentation in most tumours is between 60 and 80 yrs [4]. Even though there is no characteristic symptom complex for secondary tumours of the penis, most patients present with mass or induration of the penis. Priapism is reported with varying frequencies but is a prominent feature in nearly 40% of patients [2]. It could be caused either by occlusion of the draining veins or secondary to thrombosis in the cavernosal spaces caused by the infiltrating tumour cells [2]. Pain is not a prominent symptom in most patients and when present is localised partly to the penis and partly to the perineum [4]. Obstructive voiding symptoms and hematuria are very rarely reported [2].

In up to 60%, the metastatic lesions present as multiple infiltrative nodules [4]. Less commonly, they can be solitary nodules or ulcerative lesions, the latter mostly in the glans. Bilateral involvement of corpora cavernosa is seen in up to 65% of cases and 15% has unilateral corporal lesions. Lesions of the glans penis may be seen in 10%, though many have synchronous involvement of the corpora as well. Preputial lesions are uncommon and are usually associated with involvement of the corpora or glans [2].

### Diagnosis and treatment

Diagnosis is usually made by biopsy or corporeal aspiration, which help to differentiate between metastasis and primary tumours. Cavernosography, though useful to delineate the extent of involvement of the corporal bodies, is disadvantaged by the fact that it is invasive [11]. Metastatic lesions usually present as filling defects or structural deformities of the corporal bodies or glans. In the presence of malignant priapism, obstruction of penile venous flow can also be demonstrated. Non-invasive modalities like colour-coded duplex ultrasonography, CT scan and MRI are being increasingly used to stage the disease. Ultrasonography is operator dependant whereas imaging in only one plane limits the diagnostic value of CT. MR scanning is a reliable alternative for confirming the diagnosis and assessing the extend of the disease. On T1-weighted images, these lesions have low signal intensity, isointense with the surrounding corpus cavernosum. On T2-weighted imaging, they appear inhomogenous with low to intermediate signal intensity, seen clearly against the high background intensity of the cavernosal bodies [12].

The main differential diagnosis include primary benign and malignant penile lesions, chancre, chancroid, non-tumourous priapism, peyronie's disease, tuberculosis and non-specific inflammatory and suppurative lesions of the penis [2]. (Table 2)

**Table 2:** Differential diagnosis of metastatic penile lesions

**Pre-malignant and malignant lesions**
• Bowen's disease/Erythroplacia of Querat
• Verrucous carcinoma
• Squamous cell carcinoma
• Basal cell carcinoma
• Melanoma
• Sarcoma
**Infectious lesions**
• Tuberculosis
• Syphilitic chancre
• Chancroid
• Cryptococal infection
• Filariasis
• Rhinosporiodiosis
**Other benign conditions**
• Non-tumourous priapism
• Peyronnie's disease
• Factitous ulcerations (self induced)
• Pseudo tumours (secondary to foreign bodies or injections into penis)

The choice of treatment is greatly influenced by the general health of the patient, as well as the site of primary, extent of metastatic spread and the severity of symptoms. Most patients will require only supportive or palliative therapy. Local excision, partial or complete penectomy, external beam radiotherapy and chemotherapy have all yielded uniformly poor results. Hormonal manipulation has been tried for secondaries from prostatic adenocarcinoma, though without much success [2]. Brachytherapy can be used to deliver high local doses of radiation and has been shown to control local disease progression for up to one year [13].

### Prognosis

In general, the outlook for patients presenting with secondary malignancy in the penis is very poor, irrespective of the site of primary and the type of therapy. Most patients have widespread metastatic disease and are in a state of poor general health. Except for a few patients with small isolated lesions, which might respond to wide excision or penectomy, the majority succumb to the disease process within a year of presentation [14]. For intractable pain, total penectomy or dorsal nerve section may be indicated [15,16]. Overall patients with rectal primaries seems to fare slightly better, with two patients surviving over nine years [2]. Patients with genitourinary primary had an average survival of 47 weeks only, even with some form of treatment [14]. The longest recorded survival in this group is 7 years, for a patient with prostatic adenocarcinoma [17].

## Conclusion

Secondary malignancy of the penis is an uncommon clinical entity. However the rarity of these lesions and the varied modes of clinical presentation necessitate a good working knowledge of this condition to plan appropriate treatment in these patients. The majority arise from prostate, bladder or the recto-sigmoid and is usually associated with disseminated metastatic disease. The common mode of spread to the penis is by retrograde venous route. However, in the presented case, retrograde lymphatic spread may be a more plausible explanation for the metastasis occurring only to the penile foreskin, without involvement of the corpora or glans. The overall outcome is very poor and most patients will need only palliative or supportive care.

## Competing interests

The author(s) declare that they have no competing interests.
